# Comparison of vonoprazan with different frequency Amoxicillin regimens in the treatment of Helicobacter pylori infections: A retrospective study

**DOI:** 10.1097/MD.0000000000043998

**Published:** 2025-08-29

**Authors:** Yonggang Li, Ning Chang, Xiaomeng Han, Depeng Liang, Shan Jiang, Kun-Kun Li

**Affiliations:** a Department of Gastroenterology, Zhengzhou Central Hospital Affiliated to Zhengzhou University, Zhengzhou, China.

**Keywords:** dual therapy, frequency of administration, helicobacter pylori, Vonoprazan

## Abstract

This study aims to evaluate the efficacy of vonoprazan combined with amoxicillin (vonoprazan-amoxicillin [VA]) at the same dose but different administration frequencies in treating Helicobacter pylori (Helicobacter pylori [Hp]) infection. This retrospective study analyzed 236 Hp-positive patients. Patients were treated with the VA regimen for 14 days and divided based on amoxicillin administration frequency: L-VA: vonoprazan 20 mg bid + amoxicillin 1 g tid; H-VA: vonoprazan 20 mg bid + amoxicillin 0.75 g qid. A ^13^C or ^14^C-urea breath test was performed at least 4 weeks posttreatment to assess eradication rates and adverse events in both groups. The eradication rates of L-VA and H-VA were 85.5% and 93.8%, respectively. The eradication rate of H-VA group was higher than L-VA group, the difference between 2 groups was statistically significant (*P* = .039). The incidence of adverse events was 8.1% in the L-VA group and 7.1% in the H-VA group, but there was no significant difference in the incidence of adverse events between the groups (*P* = .258). BMI and the overall eradication rates in 2 groups showed no significant difference (*P* = .102). However, Among patients with BMI ≥ 24%, the eradication rate of H-VA group reached 100%, which significantly differed from the L-VA group (*P* = .039). Amoxicillin administered 4 times daily is more effective than 3 times daily in eradicating Hp, with an eradication rate > 90% and lower adverse events incidence, improving related discomfort symptoms. This regimen is recommended as the first-line treatment for Hp.

## 1. Introduction

Helicobacter pylori (Hp) is a prevalent infectious agent that impacts approximately 50% of the global population, with particularly high infection rates in developing nations.^[1,2]^ In certain adult demographics, infection prevalence may reach up to 90%, with a significant proportion of individuals remaining asymptomatic.^[3]^ The World Health Organization classifies Hp as a Class I carcinogen, linking it to various gastrointestinal disorders, including duodenal ulcers, gastritis, non-ulcer dyspepsia, gastric mucosa-associated lymphoid tissue lymphoma, and gastric cancer^[4]^; Consequently, clinical guidelines advocate for eradication therapy in individuals infected with Hp, regardless of symptomatology.^[5]^ In recent years, with the increase of antibiotic resistance and the difference in the effect of acid-suppressing drugs, the eradication rate of first-line treatment regimens has gradually decreased to below 90%, and the re-ignition rate and drug resistance after treatment are increasing.^[6]^ Therefore, identifying effective eradication strategies that are resilient to antibiotic resistance is imperative.

The emergence of vonoprazan provides a new option for Hp treatment. Vonoprazan is a potassium-competitive acid blocker approved in Japan in 2015 for the treatment of acid-related diseases. Currently, its clinical applications encompass gastroesophageal reflux disease, nonerosive reflux disease, erosive esophagitis, and peptic ulcer disease associated with Hp.^[7]^ Compared to proton pump inhibitors (PPIs), vonoprazan demonstrates a longer duration of acid suppression, heightened efficacy, and a prolonged therapeutic effect.^[8]^ Recent studies indicate that vonoprazan is superior to PPIs in the overall management of Hp, with the vonoprazan-amoxicillin combination (vonoprazan-amoxicillin [VA]) showcasing commendable eradication rates while minimizing adverse effects.^[9]^ Presently, clinical guidelines endorse the VA dual regimen as a first-line treatment; however, there remains a lack of consensus regarding the optimal dosing and frequency of amoxicillin.

Consequently, this study aims to investigate the impact of various high-frequency dosing regimens of amoxicillin, administered at a consistent total daily dose, on the efficacy of Hp eradication within the VA dual regimen.

## 2. Materials and methods

### 2.1. Study subjects

This retrospective study analyzed the clinical data of 236 patients diagnosed with Hp infection at Zhengzhou Central Hospital affiliated to Zhengzhou University from March 2023 to April 2024, received the VA dual therapy regimen. The inclusion criteria were as follows: age ≥ 18 years; patients diagnosed with current Hp infection through the ^13^C/^14^C urea breath test (UBT); patients assessed with the ^13^C/^14^C-UBT at least 4 weeks after discontinuing medication to confirm eradication of Hp. The exclusion criteria were as follows: age < 18 years; history of distal gastrectomy; concurrent use of histamine H_2_ antagonists or traditional Chinese medicine during Hp eradication; incomplete medical records. The study was approved by the Medical Ethics Committee of Zhengzhou Central Hospital (ZXYY2024115).

### 2.2. Diagnosis and treatment options

Prior to initiating treatment, it is essential to assess for an active Hp infection utilizing the ^13^C/^14^C-UBT. To mitigate the risk of false-negative results associated with the ^13^C/^14^C-UBT, it is crucial that patients refrain from the use of acid-suppressing medications and antibiotics for at least 2 weeks prior to undergoing the test. Following this assessment, patients will be prescribed a 14-day regimen of dual therapy with vonoprazan and amoxicillin. According to the frequency of amoxicillin administration, they were divided into 2 groups: L-VA (n = 124): vonoprazan 20 mg bid + amoxicillin 1 g tid; H-VA (n = 112): vonoprazan 20 mg bid + amoxicillin 0.75 g qid.

### 2.3. Statistical analysis

Data collected in this study were analyzed statistically using SPSS.27 (Chicago). The primary outcomes assessed included the eradication rate and the incidence of adverse events (Adverse events, AEs). Continuous variables were presented as mean *±* standard deviation and were compared using the t-test. Categorical variables were reported as absolute counts alongside percentage frequencies. To compare outcomes between the treatment groups, statistical analyses were conducted using the Chi-square test and Fisher exact test, with a significance at *P* < .05.

## 3. Results

### 3.1. General information

There was no significant difference in gender, age, height, weight, body mass index (BMI), alcohol history and smoke history between the 2 groups. The underlying diseases, related digestive tract diseases (comprised atrophic and non-atrophic gastritis, peptic ulcer disease, gastroesophageal reflux disease, gastric cancer, and various other digestive tract diseases linked to Hp.) of the patients were counted (*P* > .05). There was a statistically significant difference in Hp-related symptoms (included acid reflux, heartburn, abdominal distension, abdominal pain, nausea, altered taste sensation, belching, and other clinical manifestations related to Hp infection.) between the 2 groups (*P* = .006) (Table 1).

**Table 1:** Comparison of general data among 2 groups.

	L-VA (n = 124)	H-VA (n = 112)	*t*/*χ*^2^	*P*
Gender (n [%])				
Man	55 (44.40)	43 (38.40)	0.861	.353
Woman	69 (55.60)	69 (61.60)		
Age (year)	42.44 ± 13.26	44.21 ± 12.89	−1.033	.303
Age (year n [%])				
≥60	17 (13.70)	17 (15.20)	0.103	.748
<60	107 (86.30)	95 (84.80)		
Weight (kg)	65.20 ± 10.94	63.15 ± 11.71	1.396	.164
Drink (n [%])				
Yes	26 (21.00)	22 (19.60)	0.064	.801
No	98 (79.00)	90 (80.40)		
Smoke (n [%])				
Yes	21 (16.90)	18 (16.10)	0.032	.858
No	103 (83.10)	94 (83.90)		
BMI (n [%])				
≥24	48 (38.70)	34 (30.40)	1.811	.178
<24	76 (61.30)	78 (69.60)		
Underlying disease (n [%])				
Yes	8 (6.50)	9 (8.00)	0.221	.638
No	116 (93.50)	105 (92.00)		
Related diseases (n ([%])				
Yes	23 (18.5)	20 (17.9)	0.019	.891
No	101 (81.5)	92 (82.1)		
Associated symptoms (n [%])				
Yes	15 (12.10)	3 (2.70)	7.409	.006
No	109 (87.90)	109 (97.30)		

The underlying conditions encompassed heart disease, hypertension, and diabetes; BMI is defined as weight in kilograms divided by height in meters squared (kg/m^2^).

BMI = body mass index.

### 3.2. Eradication rates

The eradication rates of L-VA and H-VA were 85.5% (106/124) and 93.8% (105/112), respectively, and the eradication rates of the H-VA group were higher than those of the L-VA group, and there was a significant difference in the eradication rates between the 2 groups (*P* = .039), which was statistically significant, that is, amoxicillin 0.75 g was administered 4 times a day in combination with vonoprazan 20 mg twice a day. The eradication effect was satisfactory, and the eradication rate was >90%; In the ≥60-year-old population, the eradication rates of the L-VA and H-VA groups were 82.40% and 94.40%, respectively, and there was no significant difference between the 2 groups (*P* = .601).

### 3.3. Comparison of adverse events

The incidence of adverse events in the L-VA group was 8.1% and 7.1% in the H-VA group, and there was no significant difference between the 2 (*P* = .258), the main adverse events (Table 2) were abdominal pain, bloating, nausea, and abnormal sense of taste, all of which were mild symptoms. Two of the patients had occasional tinnitus symptoms during the treatment period, and all symptoms disappeared after the treatment.

**Table 2:** Treatment-related adverse events in 2 groups.

	L-VA	H-VA	*χ* ^2^	*P*
Total	10	5	1.282	.258
Abnormal sense of taste	2	0	–	–
Disgusting	2	0	–	–
Abdominal distension	3	3	–	–
Diarrhea	0	1	–	–
Bellyache/	1	1	–	–
Tinnitus	2	0	–	–

VA = vonoprazan-amoxicillin.

### 3.4. Effect of BMI and underlying diseases on the eradication rate

The eradication rate of BMI ≥24% was 93.9% (77/82), and the eradication rate of BMI <24% was 87% (134/154), and there was no significant difference in the overall eradication rate between the 2 groups (*P* = .102). Among the patients with BMI ≥24%, there were 48 patients in the L-VA group and 34 patients in the H-VA group, and the eradication rates were 87.5% and 100%, respectively, and there was a significant difference between the 2 groups (*P* = .039), which was statistically significant. Among the patients with a BMI of < 24%, there was no significant difference between the 2 (*P* = .199) (Table 3). This study showed that the VA dual regimen was not affected by BMI, but there is a significant difference between the 2 treatment plans for individuals with a BMI of 24% or higher. On the other hand, in this study, the eradication rate of Hp patients with underlying diseases was 76.5% (13/17), and no adverse events occurred, indicating that the regimen was safe, but the eradication effect was not satisfactory.

**Table 3:** Effect of BMI on eradication rate.

BMI	L-VA	H-VA	*χ* ^2^	*P*
<24%	84.2% (64/76)	91% (71/78)	1.653	.199
≥24%	87.5% (42/48)	100% (34/34)	4.586	.039

BMI is defined as weight in kilograms divided by height in meters squared (kg/m^2^).

BMI = body mass index, VA = vonoprazan-amoxicillin.

## 4. Discussion

Hp is a gram-negative microaerophilic bacterium commonly found in the human gastric mucosa, which is generally dormant and gradually proliferates actively when the pH in the stomach rises.^[10]^ In addition to its role in gastrointestinal disorders, Hp is also implicated in several extra-gastrointestinal diseases, including iron deficiency anemia and idiopathic thrombocytopenic purpura, among others.^[11]^ Studies indicate that the direct incidence of Hp causing gastric cancer is low; however, approximately 90% of gastric cancer cases are associated with Hp infection.^[12]^ In addition, Hp has also been found to be associated with colorectal adenoma.^[13]^ Therefore, the eradication of Hp can promote the healing of gastric mucosa, reduce the incidence of gastritis, and reduce the risk factors of gastric cancer, thereby reducing the medical burden and obtaining long-term health benefits. However, with a significant increase in antibiotic resistance, most current treatment regimens do not have an eradication rate of 90% or higher.^[14]^ This study found that in the VA dual regimen, amoxicillin 0.75 g 4 times a day had an eradication rate of more than 90% and a low incidence of adverse events, this intervention may yield enhanced health benefits for Hp-positive patients, with a notable improvement in symptoms observed posttreatment, particularly in relation to BMI.

On the other hand, the acid inhibition effect is a key factor affecting Hp treatment. The acid inhibition mechanism of vonoprazan is different from that of PPI, and vonoprazan is transmitted by means of H^+^-K+-ATPase binds reversibly and exerts a potent acid-suppressing effect without gastric acid activation and is not affected by ingestion.^[15]^ Research demonstrates that daily administration of 20 mg of vonoprazan can prolong the gastric pH above 4 for over 90% of the time. Following a continuous regimen of 40 mg of vonoprazan for 7 days, the duration of pH exceeding 5 can reach an impressive 100%.^[16]^ The high pH of the gastric environment is conducive to activating the proliferation and exposure of Hp, thereby enhancing the bactericidal effect of antibiotics to increase the eradication rate.^[17]^ Traditional PPIs exhibit a slow onset of action, require acid activation, and have a brief duration of effect, while their efficacy is influenced by CYP2C19 genetic polymorphisms. This variability can lead to disparate acid suppression across different populations, potentially contributing to treatment failures.^[18]^ Conversely, vonoprazan acts swiftly, necessitates no acid activation, and remains unaffected by CYP2C19 genetic variability.^[19]^ In scenarios where antibiotic resistance is uncertain and sensitivity testing is not yet widespread, vonoprazan can deliver extensive acid suppression. Additionally, amoxicillin, being both time-dependent and acid-dependent, can sustain its minimal inhibitory concentration through frequent administration in a high pH environment, thereby exerting potent bactericidal effects. The synergistic potential of vonoprazan’s robust acid-suppressive action alongside amoxicillin’s pharmacodynamic properties suggests that the VA dual therapy regimen may represent an optimal strategy for Hp treatment.

Research findings indicate that, in both randomized controlled trials and non-randomized controlled studies, the Hp eradication rate associated with vonoprazan exceeds that of PPIs.^[20]^ The results showed that although there was no significant difference in the eradication rate between the dual therapy and triple therapy, the VA dual regimen had low adverse events and reduced the use of antibiotic, indirectly reducing antibiotic resistance.^[21]^ A study in Japan showed^[22]^: the ITT analysis eradication rate of the VA dual regimen was 85.0% (95% CI 75.8%–94.2%), the eradication rate by PP analysis was 86.4% (95% CI 77.4%–95.5%). In the treatment of clarithromycin-resistant strains, the effect of the VA dual regimen was also significantly better than that of the triple regimen (92.3% vs 76.2%; *P* = .048).^[23]^ The statistical results of Japan showed^[24]^:The cure rate of the VA dual regimen for clarithromycin-resistant strains is 80% to 85%, and the specific effect needs to be further studied. In addition, the eradication rate of the dual VA regimen in retreated patients was as high as 87%.^[19]^ Therefore, in regimens utilizing vonoprazan as the acid suppressant, the VA dual therapy regimen is recommended for eradication treatment, regardless of whether undergoing treatment for the first time or not.

In this study, the incidence of adverse events was <10%, mainly manifested as mild symptoms such as abdominal distention, diarrhea, abdominal pain, and nausea, which were consistent with the results of domestic and foreign studies.^[22,25]^ Although the latest meta-analysis^[20]^ has not explicitly addressed the adverse reactions associated with tinnitus, we hypothesize that these effects may stem from medication-induced symptoms such as dizziness and headaches, or may be related to the individuals’ personal lifestyles or health conditions. This correlation warrants further exploration through prospective studies. Some studies have suggested that the eradication rate of the VA dual therapy regimen may be influenced by patient body size, with smaller patients exhibiting higher eradication rates.^[26]^ However, in this study, we did not observe a statistically significant difference in the overall eradication rates between the 2 groups concerning BMI. Notably, in patients with a BMI ≥24%, the H-VA group achieved a 100% eradication rate, which was significantly higher than the L-VA group (87.5%) with a *P*-value of .039. In patients with a BMI <24%, there was no significant difference between the 2 groups, though the H-VA group maintained an eradication rate exceeding 90%. Thus, it can be inferred that irrespective of BMI, H-VA represents a preferable treatment option, consistent with our study’s findings. Research indicates that gastric mucosal atrophy significantly increases with age, leading to a reduction in gastric acid secretion. Consequently, this phenomenon is associated with higher eradication rates in individuals aged 60 years and older.^[27]^ However, the correlation between age and the efficacy of various eradication regimens remains ambiguous.^[28]^ In the current study, no significant relationship was identified between age and eradication rates.

In recent years, researchers have explored the relationship between the eradication rate of the 10-day and 14-day VA dual regimens, and found that the eradication rates of both regimens exceeded 90%, but there was no significant statistical difference.^[9,29]^ A comprehensive analysis of the results of the study on the medication cycle of VA dual regimen showed that the eradication rates of 7 days, 10 days, and 14 days PP Analysis were: 81.6%, 88.3%, 94%, ITT analysis: 78.6%, 86.4%, 91.6%,respectively. The eradication rate of the 10-day and 14-day regimens was better than that of the 7-day regimen, but there was no significant difference between the 10-day and 14-day regimens.^[30]^ In addition, Li^[31]^ and Liu^[32]^ found that amoxicillin 0.75 g tid and vonoprazan 20 mg bid in the VA dual regimen achieved good eradication results, and the eradication rates of PP analysis were 90.63% (58/64) and 90.6% (48/53), respectively. This substantiates the feasibility of the proposed regimen, reducing the dosage of amoxicillin while achieving satisfactory eradication results. Consequently, there is a pressing need for further multicenter, large-sample studies on the dual therapy with VA in order to identify regimens that entail shorter treatment durations, lower drug dosages, and satisfactory eradication outcomes.

The limitations and disadvantages of this study are as follows: First, this study is a retrospective study with a small sample size and only includes the eradication effect of the VA dual therapy regimen on some Hp-positive patients in a city in Henan Province, with limited geographical scope. Therefore, future prospective studies with multicenter and large samples are needed for verification; Secondly, this study has not yet collected information on patient Hp resistance, compliance, and other related factors.

## 5. Conclusion

The VA dual therapy regimen, involving the administration of amoxicillin 4 times daily, is both efficacious and safe for the treatment of Hp, boasting an eradication rate exceeding 90% and AEs occurring in <10%, most of which are mild. Consequently, the H-VA dual therapy regimen is recommended for first-line treatment.

## Author contributions

**Data curation:** Yonggang Li, Shan Jiang.

**Resources:** Yonggang Li, Xiaomeng Han, Depeng Liang, Kun-Kun Li.

**Writing – original draft:** Yonggang Li.

**Writing – review & editing:** Yonggang Li.

**Supervision:** Ning Chang, Kun-Kun Li.

**Visualization:** Kun-Kun Li.

## References

[1] Chen YC Malfertheiner P Yu HT. Global prevalence of helicobacter pylori infection and incidence of gastric cancer between 1980 and 2022. Gastroenterology. 2024;166:605–19. 38176660 10.1053/j.gastro.2023.12.022

[2] Ren S Cai P Liu Y. Prevalence of helicobacter pylori infection in China: a systematic review and meta‐analysis. J Gastroenterol Hepatol. 2022;37:464–70. 34862656 10.1111/jgh.15751

[3] Salih B. Helicobacter pylori infection in developing countries: the burden for how long? . Saudi J Gastroenterol. 2009;15:201. 19636185 10.4103/1319-3767.54743 PMC2841423

[4] Al-Fakhrany OM Elekhnawy E. Helicobacter pylori in the post-antibiotics era: from virulence factors to new drug targets and therapeutic agents. Arch Microbiol. 2023;205:301. 37550555 10.1007/s00203-023-03639-0 PMC10406680

[5] Liu WZ Xie Y Lu H; Chinese Society of Gastroenterology, Chinese Study Group on Helicobacter Pylori and Peptic Ulcer. Fifth Chinese national consensus report on the management of helicobacter pylori infection. Helicobacter. 2018;23:e12475. 29512258 10.1111/hel.12475

[6] Zhou L Lu H Song Z; on Behalf of Helicobacter Pylori Study Group of Chinese Society of Gastroenterology. 2022 Chinese national clinical practice guideline on helicobacter pylori eradication treatment. Chin Med J (Engl). 2022;135:2899–910. 36579940 10.1097/CM9.0000000000002546 PMC10106216

[7] Savarino V Antonioli L Fornai M. An update of pharmacology, efficacy, and safety of vonoprazan in acid-related disorders. Expert Rev Gastroenterol Hepatol. 2022;16:401–10. 34550866 10.1080/17474124.2021.1984878

[8] Graham DY Dore MP. Update on the use of vonoprazan: a competitive acid blocker. Gastroenterology. 2018;154:462–6. 29337157 10.1053/j.gastro.2018.01.018

[9] Du R Hu Y Ouyang Y. Vonoprazan and amoxicillin dual therapy as the first‐line treatment of helicobacter pylori infection: a systematic review and meta-analysis. Helicobacter. 2024;29:e13039. 38036941 10.1111/hel.13039

[10] Cardos IA Zaha DC Sindhu RK Cavalu S. Revisiting therapeutic strategies for H. pylori treatment in the context of antibiotic resistance: focus on alternative and complementary therapies. Molecules. 2021;26:6078. 34641620 10.3390/molecules26196078 PMC8512130

[11] Aumpan N Mahachai V Vilaichone R. Management of helicobacter pylori infection. JGH Open. 2023;7:3–15. 36660052 10.1002/jgh3.12843 PMC9840198

[12] Plummer M Franceschi S Vignat J Forman D de Martel C. Global burden of gastric cancer attributable to helicobacter pylori: helicobacter pylori in gastric cancer. Int J Cancer. 2015;136:487–90. 24889903 10.1002/ijc.28999

[13] Zhuang ZM Yu BQ Xie M Zhang YG. Correlation between *Helicobacter pylori* enrichment and clinical and pathological characteristics of colorectal adenoma [in Chinese]. Zhonghua Yi Xue Za Zhi. 2022;102:3543–8. 36418254 10.3760/cma.j.cn112137-20220421-00882

[14] Bujanda L Nyssen OP Ramos J. Effectiveness of helicobacter pylori treatments according to antibiotic resistance. Am J Gastroenterol. 2024;119:646–54. 37983769 10.14309/ajg.0000000000002600

[15] Mulford DJ Leifke E Hibberd M Howden CW. The effect of food on the pharmacokinetics of the potassium – competitive acid blocker vonoprazan. Clin Pharmacol Drug Dev. 2022;11:278–84. 34431240 10.1002/cpdd.1009 PMC9291755

[16] Iwakiri K Sakurai Y Shiino M. A randomized, double-blind study to evaluate the acid-inhibitory effect of vonoprazan (20 mg and 40 mg) in patients with proton-pump inhibitor-resistant erosive esophagitis. Therap Adv Gastroenterol. 2017;10:439–51. 10.1177/1756283X17705329 PMC5424876 28567114

[17] Graham DY Liou JM. Primer for development of guidelines for helicobacter pylori therapy using antimicrobial stewardship. Clin Gastroenterol Hepatol. 2022;20:973–83.e1. 33775895 10.1016/j.cgh.2021.03.026 PMC8464630

[18] Abadi ATB Ierardi E. Vonoprazan and helicobacter pylori treatment: a lesson from Japan or a limited geographic phenomenon? . Front Pharmacol. 2019;10:316. 31024299 10.3389/fphar.2019.00316 PMC6459936

[19] Katayama Y Toyoda K Kusano Y. Efficacy of vonoprazan-based second-line helicobacter pylori eradication therapy in patients for whom vonoprazan-based first-line treatment failed. Gut. 2017;66:752–3. 27196582 10.1136/gutjnl-2016-312028

[20] Jiang Y Zhang R Fang Y. P-CAB versus PPI in the eradication of helicobacter pylori: a systematic review and network meta-analysis. Therap Adv Gastroenterol. 2024;17:17562848241241223. 10.1177/17562848241241223 PMC11095192 38751605

[21] Fallone CA. The current role of vonoprazan in helicobacter pylori treatment. Gastroenterology. 2022;163:572–4. 35780869 10.1053/j.gastro.2022.06.076

[22] Gotoda T Kusano C Suzuki S Horii T Ichijima R Ikehara H. Clinical impact of vonoprazan-based dual therapy with amoxicillin for H. Pylori infection in a treatment-naïve cohort of junior high school students in Japan. J Gastroenterol. 2020;55:969–76. 32666199 10.1007/s00535-020-01709-4

[23] Suzuki S Gotoda T Kusano C. Seven-day vonoprazan and low-dose amoxicillin dual therapy as first-line *Helicobacter pylori* treatment: a multicentre randomised trial in Japan. Gut. 2020;69:1019–26. 31915235 10.1136/gutjnl-2019-319954 PMC7282559

[24] Graham DY Lu H Shiotani A. Vonoprazan‐containing helicobacter pylori triple therapies contribution to global antimicrobial resistance. J Gastroenterol Hepatol. 2021;36:1159–63. 32918832 10.1111/jgh.15252

[25] Liu Z Chen X Sun DJ. Comparison of vonoprazan-based dual therapy with vonoprazan-based bismuth quadruple therapy for treatment-naive patients with helicobacter pylori infection: a propensity score matching analysis. Medicine (Baltimore). 2024;103:e37476. 38457567 10.1097/MD.0000000000037476 PMC10919513

[26] Eto H Suzuki S Kusano C. Impact of body size on first-line helicobacter pylori eradication success using vonoprazan and amoxicillin dual therapy. Helicobacter. 2021;26:e12788. 33580612 10.1111/hel.12788

[27] Saito Y Konno K Sato M. Vonoprazan-based third-line therapy has a higher eradication rate against sitafloxacin-resistant helicobacter pylori. Cancers. 2019;11:116. 30669474 10.3390/cancers11010116 PMC6356600

[28] Kusunoki M Yuki M Ishitobi H. Effect of age on effectiveness of vonoprazan in triple therapy for *Helicobacter pylori* eradication. Intern Med. 2019;58:1549–55. 30713328 10.2169/internalmedicine.2233-18 PMC6599932

[29] Peng X Yao JY Ma YQ. Efficacy and safety of vonoprazan-amoxicillin dual regimen with varying dose and duration for helicobacter pylori eradication: a multicenter, prospective, randomized study. Clin Gastroenterol Hepatol. 2024;22:1210–6. 38309492 10.1016/j.cgh.2024.01.022

[30] Du RC Ouyang YB Lu NH Hu Y. Research trends on vonoprazan-based therapy for helicobacter pylori eradication: a bibliometric analysis from 2015 to 2023. Helicobacter. 2023;28:e13012. 37515414 10.1111/hel.13012

[31] Li J Lv L Zhu Y Zhou Z He S. A modified 14-day dual therapy with vonoprazan and amoxicillin amplified the advantages over conventional therapies for eradication of helicobacter pylori: a non-inferiority clinical trial. Infect Drug Resist. 2023;16:5637–45. 37662977 10.2147/IDR.S417711 PMC10473400

[32] Liu Z Sun D Kou L. Vonoprazan-amoxicillin dual therapy with different amoxicillin dosages for treatment-naive patients of helicobacter pylori infection in China: a prospective, randomized controlled study. Eur J Gastroenterol Hepatol. 2024;36:712–9. 38526917 10.1097/MEG.0000000000002760

